# Venue-Based HIV Testing at Sex Work Hotspots to Reach Adolescent Girls and Young Women Living With HIV: A Cross-sectional Study in Mombasa, Kenya

**DOI:** 10.1097/QAI.0000000000002363

**Published:** 2020-04-08

**Authors:** Huiting Ma, Linwei Wang, Peter Gichangi, Vernon Mochache, Griffins Manguro, Helgar K. Musyoki, Parinita Bhattacharjee, François Cholette, Paul Sandstrom, Marissa L. Becker, Sharmistha Mishra

**Affiliations:** a MAP-Centre for Urban Health Solution, St. Michael's Hospital, Unity Health Toronto, Toronto, Canada;; b Department of Human Anatomy, University of Nairobi, Nairobi, Kenya;; c International Centre for Reproductive Health-Kenya, Mombasa, Kenya;; d University of Maryland, Centre for International Health, Education and Biosecurity, College Park, MA;; e National AIDS & STI Control Programme, Nairobi, Kenya;; f Key Populations Technical Support Unit, Partners for Health and Development in Africa, Nairobi, Kenya;; g Centre for Global Public Health, University of Manitoba, Winnipeg, Canada;; h National HIV and Retrovirology Laboratory, JC Wilt Infectious Diseases Research Centre, Public Health Agency of Canada, Winnipeg, Canada;; i Department of Medical Microbiology and Infectious Diseases, University of Manitoba, Winnipeg, Canada;; j Department of Medicine, University of Toronto, Toronto, Canada;; k Institute of Medical Science, University of Toronto, Toronto, Canada; and; l Institute of Health Policy, Management, and Evaluation, University of Toronto, Toronto, Canada.

**Keywords:** sex work, adolescent girls and young women, HIV testing, hotspots, HIV cascade

## Abstract

We estimated the potential number of newly diagnosed HIV infections among adolescent girls and young women (AGYW) using a venue-based approach to HIV testing at sex work hotspots.

**Methods:**

We used hotspot enumeration and cross-sectional biobehavioral survey data from the 2015 *Transition Study* of AGYW aged 14–24 years who frequented hotspots in Mombasa, Kenya. We described the HIV cascade among young females who sell sex (YFSS) (N = 408) versus those young females who do not sell sex (YFNS) (N = 891) and triangulated the potential (100% test acceptance and accuracy) and feasible (accounting for test acceptance and sensitivity) number of AGYW that could be newly diagnosed through hotspot-based HIV rapid testing in Mombasa. We identified the profile of AGYW with an HIV in the past year using generalized linear mixed regression models.

**Results:**

N = 37/365 (10.1%) YFSS and N = 30/828 (3.6%) YFNS were living with HIV, of whom 27.0% (N = 10/37) and 30.0% (N = 9/30) were diagnosed and aware (*P* = 0.79). Rapid test acceptance was 89.3%, and sensitivity was 80.4%. There were an estimated 15,635 (range: 12,172–19,097) AGYW at hotspots. The potential and feasible number of new diagnosis was 627 (310–1081), and 450 (223–776), respectively. Thus, hotspot-based testing could feasibly reduce the undiagnosed fraction from 71.6% to 20.2%. The profile of AGYW who recently tested was similar among YFSS and YFNS. YFSS were 2-fold more likely to report a recent HIV test after adjusting for other determinants [odds ratio (95% confidence interval): 2.2 (1.5 to 3.1)].

**Conclusion:**

Reaching AGYW through hotspot-based HIV testing could fill gaps left by traditional, clinic-based HIV testing services.

## Abstract 2

Supplemental Digital Content is Available in the Text.

## INTRODUCTION

Adolescent girls and young women (AGYW) aged 15–24 years face a disproportionate risk of HIV acquisition in sub-Saharan Africa (SSA).^1^ In Kenya, AGYW comprise 18.4% of the adult population but acquired 23.7% of new infections in 2017, such that, by 2018, an estimated 2.6% of AGYW in Kenya were living with HIV^2–5^; yet, most infections remain undiagnosed.^4^ The most recent data available on AGYW suggest that, in 2012, only 25% of AGYW living with HIV were diagnosed and aware of their HIV status.^4^ The consequence of undiagnosed HIV among AGYW is untreated HIV, thus limiting the individual health and the population-level transmission benefits of effective antiretroviral therapy (ART).^6–8^

HIV testing serves as an entry point for HIV care with a growing recognition that differentiated strategies^9,10^—that is, services tailored to subgroups within a population—are needed to address subgroup-specific barriers to traditional, clinic-based testing.^10,11^ For example, service-related barriers reported by adolescents in SSA include stigma from health care providers and logistical challenges, such as costs and time for transportation to and from clinics whose hours of operation often conflict with school or employment.^11,12^ Although data on differentiated strategies to improve HIV testing among AGYW remain limited,^9,10^ emerging evidence suggests that venue-based testing, under the umbrella of community-based approaches, may be an effective strategy to increase HIV testing among subgroups at high risk of HIV.^13–15^

Venues refer to places where a particular subgroup may uniquely come together and socialize (schools, shopping malls, and parks) and/or where people meet new sex partners.^16^ For example, Herce et al^13^ found that venue-based testing and counseling conducted as part of a survey of female sex workers, led to the new diagnosis of 63% of those living with HIV but who were previously unaware of their HIV status. Venues associated with formal sex work, or sex work hotspots, are also places where AGYW, including young females who sell sex (YFSS), congregate, socialize, and meet sex partners. For example, in Mombasa, Kenya, 95% of hotspots comprise venues where AGYW not engaged in sex work socialize and meet sex partners.^16^ Some of these young women engage in other forms of transactional sex or casual sex and experience high prevalence of HIV-associated vulnerabilities at first sex, similar to the prevalence reported by YFSS.^17^

The age of consent for HIV testing in Kenya is 15 years.^18^ As in most of SSA, existing HIV testing programs in Kenya are designed for the wider population of AGYW, and/or they are designed to reach formal sex workers; they are not specifically designed to reach high-risk AGYW such as those who socialize in hotspots.^18,19^ Similarly, most population-based studies on HIV testing in SSA are often conducted separately for AGYW (usually through household surveys) and for female sex workers (usually restricted to those over age 18 years).^20–23^ Thus, there are limited data on HIV testing patterns and undiagnosed HIV among high-risk AGYW and YFSS who socialize in the same spaces. Yet, studies conducted separately in each population (AGYW, female sex workers) suggest determinants of HIV testing uptake may be similar.^20–22,24–26^

Limited data suggest YFSS face similar service-related barriers to testing programs designed for adults as those reported by the wider population of AGYW; barriers are compounded by stigma and logistical challenges related to sex work and which may also undermine access to programs designed for AGYW in general, such as school-based testing.^11,22,27,28^ Meanwhile, YFSS are often excluded from sex worker programs, which provide or facilitate clinic-based HIV testing, but are designed to serve women aged 18 and over who self-identify as sex workers.^23,29^ Currently, in Kenya, HIV testing does not include venue-based testing at hotspots,^18^ and before 2018, sex worker programs were not allowed to provide services for women under age 18.^19,30^ The consequence of vertical programs and independently studied populations is that we do not yet know the potential value of venue-based testing at hotspots for AGYW, and whether determinants of HIV testing differ between YFSS and other AGYW who frequent the same hotspots.

Among AGYW who frequent hotspots in Mombasa, Kenya, we sought to (1) estimate the number of AGYW living with HIV that could be newly diagnosed through hotspot-based testing and (2) identify determinants of recent HIV testing among AGYW who frequent hotspots.

## METHODS

### Study Setting and Population

We used data from hotspot enumeration and the *Transition* Study, cross-sectional biobehavioral survey of AGYW recruited at hotspots in Mombasa, Kenya, from April to November 2015.^16,17^ Survey eligibility included cis-gender female aged 14–24 years who reported engaging in vaginal or anal sex at least once in their lifetime.

### Data Collection

We conducted mapping and enumeration of hotspots before survey implementation to estimate the number of AGYW aged 14–24 years congregating at hotspots and to generate the sampling frame as detailed in Cheuk et al.^16^ We used probability proportional to estimated size of the AGYW population for sampling and thus generated a self-weighted sample.^16,31^ Within each sampled hotspot, outreach workers or a peer-educator invited potential participants, and trained interviewers screened for eligibility and administered a face-to-face structured questionnaire in English or Kiswahili. Participants were offered rapid, onsite HIV testing and counseling, which was administered as per Kenya national guidelines (see Supplemental Digital Content 1, http://links.lww.com/QAI/B461).^18^ We also collected dried blood specimens (DBS, detailed protocol provided in Supplemental Digital Content 2, http://links.lww.com/QAI/B461). Participants provided written informed consent with the option to consent or decline to participate in any component of the study.^17^ Data collection procedures are detailed in Becker et al.^17^

### Measures

We classified participants as YFSS if they self-identified as a sex worker or reported ever soliciting and receiving money, gifts, or other goods in exchange for sex, such that the price or commodity was negotiated before sex and as young females who do not sell sex (YFNS) otherwise. We used the DBS serology results to identify persons living with HIV. Participants without a DBS were excluded from our analyses of HIV cascade of care.

We defined the early stages of the HIV cascade among those living with HIV as follows: (1) HIV diagnosed and aware if participants self-reported as “HIV-positive” (those who self-reported negative or not willing to disclosure or never tested for HIV were classified as undiagnosed); (2) linkage to HIV care (self-reported registration with an HIV treatment center); and (3) currently on ART (self-reported they were currently taking antiretroviral medication).

We defined recent HIV testing based on self-reported HIV testing with receipt of result in the year before the survey and ever, respectively.

We defined covariates (see Supplemental Digital Content 3, http://links.lww.com/QAI/B461) to identify determinants of HIV testing as informed by previous literature^18,20–22,24,25,27^ with a focus on sociodemographic, health system engagement, sexual behavior, and risk perception, and based on data availability.

### Statistical Analyses

First, we compared the early stages of the HIV cascade including HIV diagnosed and aware, linkage to HIV care, and currently on ART, among YFSS versus YFNS living with HIV.

Second, we conducted a triangulation exercise to estimate the potential number of AGYW living with HIV in Mombasa that could be newly diagnosed through hotspot-based testing if we assumed 100% test acceptance and accuracy. We used the estimated population size of AGYW who frequent hotspots in Mombasa from the 2014 mapping and enumeration^32^; and estimates of HIV prevalence and undiagnosed fraction from the current study. To estimate the feasible number of AGYW that could be newly diagnosed, we applied plausibility constraints: acceptance of rapid testing by participants who did not self-report HIV-positive (measured as the proportion of participants who agreed and received rapid test when the test was offered) and the sensitivity of the rapid test against DBS results (as measured among those who received both rapid and DBS tests). We reported the potential and feasible estimates for the overall AGYW population in Mombasa who frequent at hotspots and separately for YFSS and YFNS. We also repeated our analyses by assuming participants living with HIV who declined to disclose their HIV status were aware of their status.

Third, we compared the proportion recently tested and patterns of HIV testing among YFSS versus YFNS. Analyses of recent HIV testing in the past year excluded participants who self-reported as “HIV-positive” and were diagnosed with HIV more than 1 year before the survey. We compared categorical variables using the χ^2^ tests or fisher's exact tests as appropriate and compared continuous variables using Kruskal–Wallis tests.

We used generalized linear mixed regression models with a logit link and a binomial distribution to identify determinants of HIV testing. To address within-cluster correlation, a hotspot-specific random intercept was specified in the model.^33^ We first explored the relationship between recent testing and covariates (see Supplemental Digital Content 3, http://links.lww.com/QAI/B461) among YFSS and among YFNS separately. To identify determinants of recent testing among AGYW who could be potentially reached by hotspot-based testing, irrespective of engagement in sex work, we performed bivariate and multivariable regression on the full sample of participants. We reported the crude odds ratio and adjusted odds ratio (AOR) with 95% confidence interval (95% CI) and restricted tests of differences to variables ≥10 respondents in each cell of a predictor-outcome table.

All statistical analyses and figures were executed using R version 3.4.2.

### Ethics

The study received ethics approval (see Supplemental Digital Content 4, http://links.lww.com/QAI/B461) from the Human Research Ethics Board at the University of Manitoba, Canada (HS16557); the Kenyatta National Hospital-University of Nairobi Ethical Review Committee, Kenya (P497/10/2017); and a research permit from the National Commission for Science, Technology and Innovation, Kenya.

## RESULTS

### Undiagnosed HIV and the HIV Cascade

Of the 1299 participants who consented to the interview (see Table 1A, Supplemental Digital Content 5, http://links.lww.com/QAI/B461), 1193 (91.8%) had DBS samples available. Participants without DBS tests were more likely to be YFSS (*P* = 0.038) and currently receiving formal education (*P* = 0.008) but were otherwise similar to those with DBS tests. Of those with a DBS test (N = 1193), 67 (5.6%) tested HIV-positive overall. The HIV prevalence was 10.1% (37/365) among YFSS and 3.6% (30/828) among YFNS (*P* < 0.001).

**FIGURE 1.**
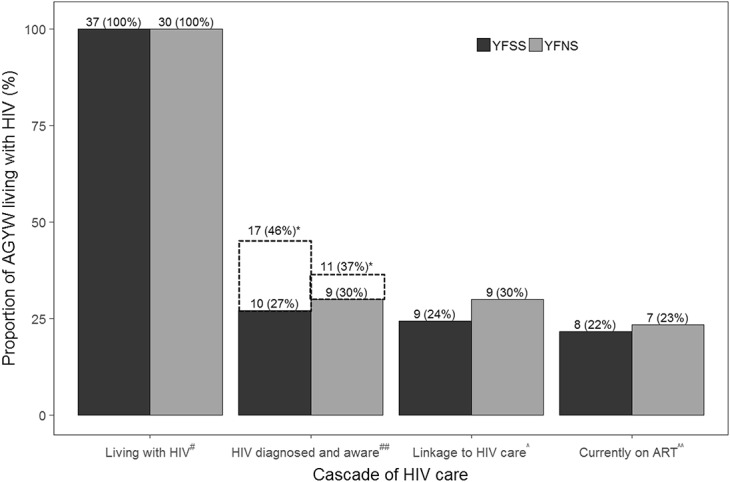
Cascade of HIV care among AGYW aged 14–24 years living with HIV by engagement in sex work in Mombasa, Kenya (N = 67). ART, antiretroviral therapy; YFNS, young females who does not sell sex; YFSS, young females who sell sex. ^#^Based on DBS serology results. ^##^Self-reported as “HIV-positive” (primary analysis: Those who self-reported as HIV-negative or not willing to disclosure or never tested for HIV were classified as undiagnosed). ^^^Self-reported registration with an HIV treatment center. ^^^^Self-reported that they were currently taking antiretroviral medication. *Sensitivity analysis: Based on the assumption that participants who were not willing to disclose their HIV status were living with HIV and were aware of their status.

Figure 1 depicts the HIV cascade. Of the 67 AGYW living with HIV, 28% (N = 19) disclosed that they were diagnosed with HIV before the interview; the proportion of diagnosed and aware was 27.0% (10/37) and 30.0% (9/30) for YFSS and YFNS, respectively (*P* = 0.79). Among those who were diagnosed, the majority of YFSS (8/10; 80.0%) and YFNS (7/9; 77.8%) self-reported to be currently on HIV treatment. A total of 13% (N = 9; YFSS: N = 7; YFNS: N = 2) of AGYW living with HIV declined to tell the interviewer their HIV status, all of whom reported an HIV test in the past year. If participants who refused to report their HIV status are assumed to be diagnosed and aware, then the proportion of diagnosed and aware would represent 46.0% (17/37) and 37.0% (11/30) of YFSS and YFNS, respectively, living with HIV (*P* = 0.44).

### Acceptance and Sensitivity of Rapid Test

A total of 1156 participants accepted rapid testing, of whom 1124 also submitted a DBS. Using the DBS results as the gold standard, the sensitivity and specificity of the rapid test algorithm were 80.4% (95% CI): 66.9 to 90.2) and 99.9% (95% CI: 99.5 to 100.0), respectively. Among those who self-reported to be HIV-negative/not willing to disclose/never tested for HIV (N = 1271), 89.3% (95% CI: 87.5 to 91.0) accepted to have rapid testing conducted.

### Number of AGYW Living With HIV Who Could be Diagnosed Through Hotspot-Based Programs

**FIGURE 2.**
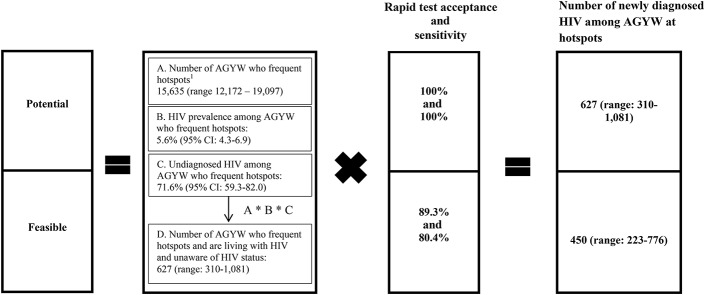
Triangulating the number of AGYW living with HIV who could be diagnosed through hotspot-based HIV testing strategy in Mombasa, Kenya. ^1^Cheuk E, Isac S, Musyoki H, et al. Informing HIV prevention programs for adolescent girls and young women: A modified approach to programmatic mapping and key population size estimation. *JMIR Public Health Surveill.* 2019; 5(2):e11196.

The estimated number of AGYW frequenting hotspots in Mombasa was 15,635 (range: 12,172–19,097), of whom an estimated 6127 (range: 4793–7462) were YFSS (Figure 2 and see Figure 4A, Supplemental Digital Content 5, http://links.lww.com/QAI/B461).^32^ Thus, using the overall HIV prevalence [5.6% (95% CI: 4.3 to 6.9)] and undiagnosed HIV fraction [71.6% (95% CI: 59.3 to 82.0)] estimates of AGYW in our study, there are an estimated 876 (range: 523–1318) AGYW living with HIV who frequent hotspots in Mombasa, among whom an estimated 627 (range: 310–1081) were undiagnosed. Therefore, the potential number of AGYW who could be newly diagnosed was 627 (range: 310–1081), and the feasible number (with 89.3% test acceptance and 80.4% sensitivity) who could be newly diagnosed was 450 (range: 223–776). If we assume participants who were living with HIV but who declined to disclose their HIV status were diagnosed and aware, the potential and feasible number who could be newly diagnosed was 510 (range: 238–925) and 366 (range: 171–664), respectively. Thus, hotspot-based testing could feasibly reduce the undiagnosed fraction among AGYW in hotspots from 71.6% (95% CI: 59.3 to 82.0) to 20.2% (95% CI: 17.6 to 23.0).

When we stratified our triangulation by engagement in sex work, the potential and feasible numbers who could be newly diagnosed were 452 (range: 193–881) and 313 (range: 134–610), respectively, among YFSS (see Figure 2A, Supplemental Digital Content 5, http://links.lww.com/QAI/B461). Among YFNS, the potential and feasible numbers who could be newly diagnosed were 240 (ranges: 93–506) and 175 (ranges 68–369), respectively (see Figure 3A, Supplemental Digital Content 5, http://links.lww.com/QAI/B461).

### Profile of AGYW and Patterns of HIV Testing in the Past Year

After excluding, 10 participants diagnosed with HIV >1 year before the survey, and 1289 were included into our analysis on patterns of recent HIV testing (Table 1). The median age was 19 years (interquartile range 17–21). Of the included participants, 81.0% were not aware of HIV services (74.0% YFSS vs. 84.2% YFNS, *P* < 0.001), and less than 1 in 10 (9.3%), AGYW were contacted by or registered with a nongovernmental or community-based organization that provides HIV prevention services. Among those with a previous HIV test, nearly all (92.6% YFSS vs. 85.8% YFNS, *P* = 0.009) said their last test was at a public or government facility.

**TABLE 1.**
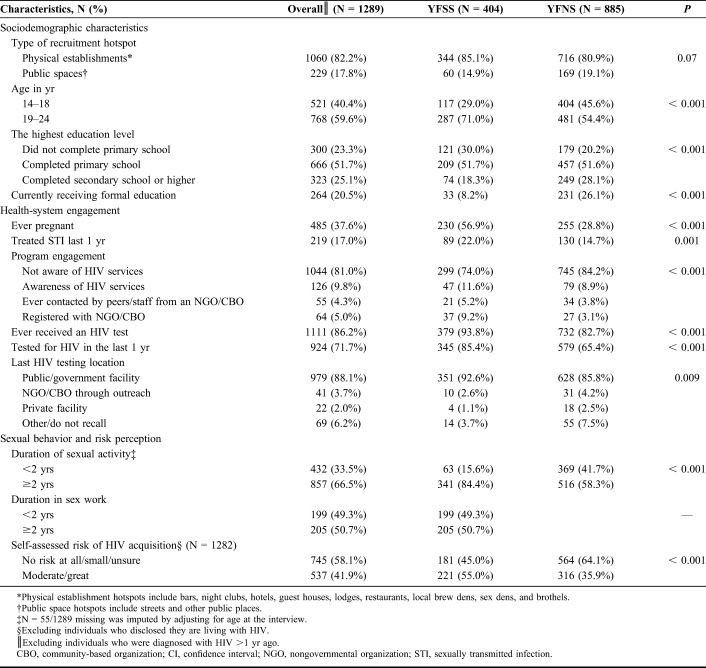
Characteristics of the Study Participants Aged 14–24 Years by Engagement in Sex Work in Mombasa, Kenya (N = 1289)

A total of 71.7% of participants reported a HIV test in the past year: 85.4% of YFSS and 65.4% of YFNS (*P* < 0.001). HIV testing frequency in the past year was also higher among YFSS than YFNS. Among YFSS and YFNS who received an HIV test in the past year, 42.3% (146/345) and 26.6% (154/579) reported having at least 2 tests in the past year (*P* < 0.001), respectively.

### Determinants of Recent HIV Testing Among AGYW Who Frequent Hotspots

#### Determinants of Recent HIV Testing Were Similar Among YFSS and YFNS

Table 3 provides the determinants of recent HIV testing among AGYW who frequent hotspots. The size and direction of determinants identified in bivariate analysis persisted after adjusting for engagement in sex work and other covariates (Tables 2 and 3). Older age [AOR (95% CI): 1.5 (1.2 to 2.1)], higher education attainment [AOR (95% CI): 1.6 (1.2 to 2.2)], and longer duration of sexual activity [AOR (95% CI): 1.4 (1.0 to 1.9) were independently associated with receiving an HIV test in the past 1 year (Table 3). Previous engagement with the health care system due to a history of pregnancy or treatment for a sexually transmitted infection in the past year were also independently associated with HIV testing [AOR (95% CI): 1.8 (1.3 to 2.5), AOR (95% CI): 1.9 (1.3 to 2.9), respectively] and awareness of sex worker programs [AOR (95% CI): 1.7 (1.2 to 2.5)]. By contrast, participants who were in formal education at the time of the survey (vs. being out of school) were less likely to have been tested in the past year [AOR (95% CI): 0.7 (0.5 to 1.0)] (Table 3). After adjusting for these determinants of recent HIV testing, YFSS compared with YFNS were two-fold more likely to have tested for HIV in the past year [AOR (95% CI): 2.2 (1.5 to 3.1)]. Compared with participants who reported they felt at no risk of HIV, those who reported they were at moderate or great risk of HIV were more likely to report a recent HIV test, but this association was no longer evident after adjusting for other determinants (Table 3).

**TABLE 2.**
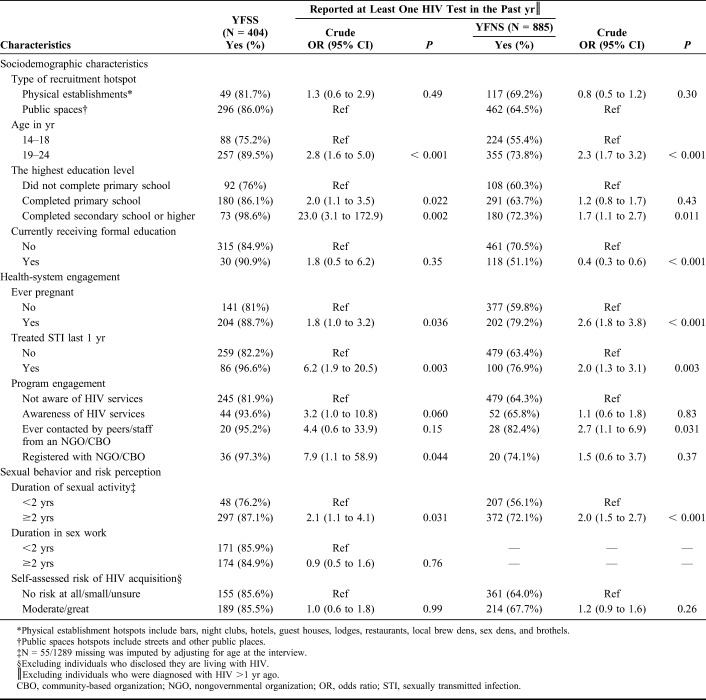
Factors Associated With HIV Testing in the Past Year Among Adolescent Girls and Young Women Aged 14–24 Years by Engagement in Sex Work in Mombasa, Kenya (N = 1289)

**TABLE 3.**
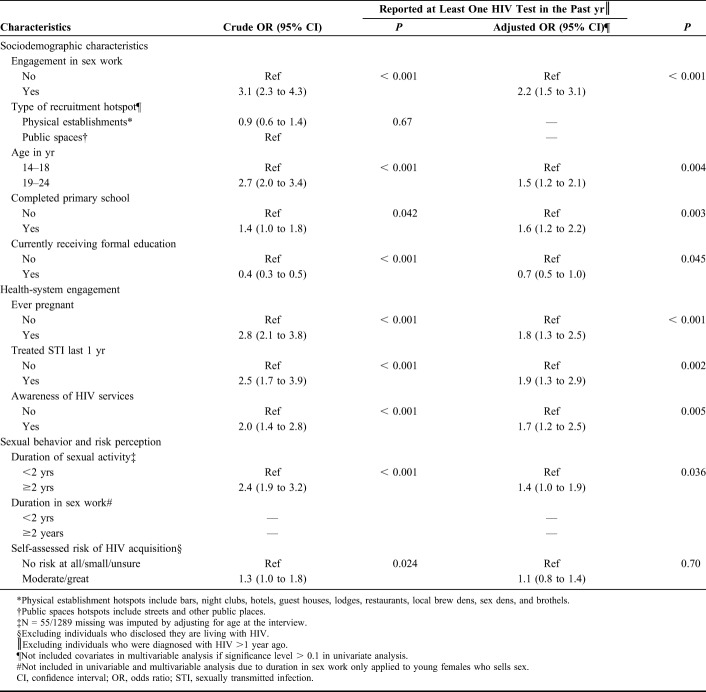
Univariate and Multivariable Analyses of Factors Associated With HIV Testing in the Past Year Among Adolescent Girls and Young Women Aged 14–24 Years in Mombasa, Kenya (N = 1289)

## DISCUSSION

We identified a large unmet need in HIV diagnoses among AGYW who frequent hotspots in Mombasa, Kenya. Although 86% of AGYW reported a lifetime history of HIV testing, only 72% were tested in the previous year, and less than 1 in 3 AGYW living with HIV were diagnosed and aware of their status. YFSS were more likely to be living with HIV and were three-fold more likely to test for HIV in the past year, and would do so more frequently, than YFNS. However, the prevalence of undiagnosed HIV and the determinants of HIV testing were similar across AGYW irrespective of whether or not they were engaged in sex work. Applying a hotspot-based strategy of onsite HIV testing with existing rapid tests could realistically and newly diagnose 51.4% of AGYW living with HIV who socialize at hotspots.

Our findings suggest that hotspots comprise subsets of AGYW with disproportionately high risk of HIV and poor access and/or uptake of HIV testing services and similar to findings of disproportionate risks among AGYW who socialize at other types of venues (bars, hotels, and transportation hubs) in East Africa.^34^ Just over one-third of YFNS at hotspots reported they felt at moderate or great risk of HIV acquisition, yet only 65.4% had ever tested for HIV and only 82.7% reported testing in the past year. The HIV prevalence among AGYW overall in Kenya was 2.8% in 2015,^35^ and thus, YFNS at hotspots may have a higher HIV prevalence than AGYW in general. We also found that YFSS and YFNS recruited from hotspots shared several determinants of HIV testing, which means that if a hotspot-based testing strategy in Mombasa was to also deploy risk-profiling to prioritize those least likely to have tested recently, it could use the same profiles for YFSS and for YFNS. Taken together, the findings support the importance of engaging AGWY who do not sell sex through hotspot-based HIV testing.

The discrepancy between the relatively high proportion of participants recently tested for HIV, yet low proportion diagnosed may reflect inadequate frequency and timing of tests in relation to changes in HIV risk over time or age. Local programs in Kenya offer HIV testing every 3 months for sex workers and annual testing for AGYW in general.^18,36^ In our study, only 9.3% of YFSS who tested in the past year did so at least 4 times; thus, most YFSS tested less frequently than what is recommended for women engaged in sex work.^18,19^ The optimal frequency and timing of tests may also need to be adapted to the changing experiences and exposures in an AGYW's sexual life course and should be facilitated by approaches that enhance an individual's agency over testing—such as HIV self-testing.^9^ In our study, 10% of YFSS were already living with HIV and yet had only been in sex work for a median of 2 years^17^—suggesting either a high prevalence of HIV before entering sex work and/or high incidence of HIV within the first 2 years of sex work. The latter in particular means that testing frequency may need to be even higher during the early period of sex work.

High levels of recent HIV testing and high undiagnosed fraction could also result from the sensitivity (81%) of the rapid tests used in the Kenya national standard protocols. Reasons for false negative results include unmeasured and field operational issues,^37^ and highlight a broader need to enhance field training, retraining, and quality assurance of rapid HIV testing as part of the national testing protocol.^38^ If we apply the false-negative rate of the rapid test to AGYW living with HIV tested in the last year, the undiagnosed fraction is still high at 62.7%. Therefore, the moderate level of sensitivity would not explain the discrepancy between high levels of previous testing and undiagnosed HIV. The discrepancy between recent testing and undiagnosed fraction is also important in the context of evaluating HIV-testing strategies, many of which use test uptake as the main outcome.^25,39^ Thus, our findings suggest that monitoring and evaluation of testing strategies should also measure undiagnosed fraction at the population level rather than just the proportion tested in the previous year.

To date, venue-based strategies deployed for AGYW have been restricted to mobile-outreach at parks and entertainment venues, all of which suggest increased uptake of HIV testing among adolescents.^14,15,40–42^ Our findings suggest hotspot-based testing strategies, such as that deployed as part of the *Transition* Study, represent an untapped opportunity to increase HIV diagnoses among AGYW living with HIV. Indeed, a population-based strategy to deliver testing services to hotspots may not require individuals to self-identify as engaging in sex work and thus provide an avenue to converge outreach and service delivery from the disparate pillars of adolescent and sex work programs. Recommendations for testing—across key and other priority populations such as AGYW—include the provision of a “safe space,” testing free of coercion and employing approaches that address stigma and discrimination related to sex work in general and to sexual activity among youth.^43^

As shown with other populations within Kenya, once diagnosed with HIV, the proportion of AGYW in our study who go on to receive ART is high,^44^ suggesting that diagnosis is the critical gap in the HIV cascade. But for hotspot-based testing strategies to also serve an entry point or HIV care, strategies to facilitate linkage to care may be needed. Potential linkage-to-care strategies that go beyond immediate referral for care include same-day ART initiation^45^ and peer navigation to support linkage,^46^ especially strategies that could leverage existing venue-based outreach by sex worker programs to facilitate testing and linkage to care for all newly diagnosed AGYW who frequent hotspots.^46^

Study limitations include the use of self-reported data collected through face-to-face interviews, which may be prone to measurement and social desirability bias, respectively. Estimates of the cascade of HIV care are also limited by the 16% of participants without reference DBS tests. Limitations on restricting our study population to those with DBS may be mitigated by the similar profile of participants with and without DBS. Thirteen percent of AGYW living with HIV who did not wish to disclose their status to the interviewer, but may have been diagnosed and aware, and thus, we may have overestimated the undiagnosed fraction. A related limitation is that we did not test for HIV-1 viral load and antiretroviral metabolites to ensure that the “new diagnoses” were not already receiving ART as conducted in other studies.^47^ To partially address this issue of ascertainment bias, we performed sensitivity analyses to obtain a lower bound estimate of the number of new HIV diagnoses.

In conclusion, there remains a large unmet need in the early elements of the HIV cascade among a particularly high-risk subset of AGYW in Kenya. Reaching AGYW through hotspot-based HIV testing strategies may reach higher risk AGYW and fill gaps left by traditional HIV prevention and testing services.

## Supplementary Material

SUPPLEMENTARY MATERIAL
