# Supplementary material for: Venue-Based HIV Testing at Sex Work Hotspots to Reach Adolescent Girls and Young Women Living With HIV: A Cross-sectional Study in Mombasa, Kenya
Source: J Acquir Immune Defic Syndr. 2020 Apr 8;84(5):470–9. doi: 10.1097/QAI.0000000000002363 (PMC7340222; doi:10.1097/QAI.0000000000002363)
Supplement: SUPPLEMENTARY MATERIAL [file qai-84-470-s001.docx]

**Supplementary Digital Content 1: Algorithm for rapid HIV testing at sex work venues (hotspots)**

A trained clinical officer/nurse counsellor performed the tests along with pre- and post-test counselling, and service-referral in accordance with the Ministry of Health of Kenya.^2^ The rapid tests were performed at or near the hotspot in a closed (confidential) room and at the same place as the face-to-face interview.

The first test was KHB HIV (1+2) Antibody (Colloidal Gold) Rapid Test (Shanghai Kehua Bio-engineering Co., Ltd, Shanghai, China); the second test was First Response HIV 1-2-O Rapid Whole Blood Test (Premier Medical Corporation Private Limited, Mumbai, India); and the tie-breaker test was Uni-Gold™ HIV Test (Trinity Biotech Plc, Bray, Ireland). If the results of first test were negative, the participant was informed of the negative results and was referred to the appropriate HIV prevention services. If the results of first test were positive, the second test was performed. If the results of second test were also positive, the participant was informed of the positive results and was referred to the appropriate HIV treatment services. If the results of second test were negative, the results were classified as discordant and a third blood sample was collected for the tie-breaker test. Participants were informed of their rapid test results at the same visit. If participants were indeterminate on rapid testing, they were referred for confirmatory testing.

Participants newly diagnosed with HIV on the confirmatory rapid test, and those preciously diagnosed but not yet linked to care, were immediately provided with a referral letter by the peer-educators to nearby government health facilities which provide HIV treatment and care free of cost to patients. Thereafter, as part of peer-based services provided by the local program (International Centre for Reproductive Health Kenya), peer-educators contacted participants referred to HIV care after the survey to check if participants received and/or used HIV services.

**Supplementary Digital Content 2: Dried Blood Spot Specimen Collection Process**

Dried blood specimens (DBS) were collected and transferred to the National HIV and Retrovirology Laboratories in Winnipeg, Canada, which performed the serological testing with the Avioq HIV-1 Microelisa System (Avioq Inc., Research Triangle Park, NC). If they were HIV-positive on DBS, but HIV-negative on rapid testing, they were not informed as we had no way to reach them.

We did not include a study-specific procedure for following up with participants after the survey. Because the study followed Kenya National guidelines for rapid HIV testing, and because serological testing of DBS would only take place after all data collection were completed (i.e. 3-4 months), we did not include a routine process for participants to receive their DBS serological results unless the participants chose to do so by contacting the study team. That is, in the consent process, we informed potential participants that the additional blood samples they may agree (or decline) to provide for DBS would be tested after study completion, and that they would not be receiving the results of the DBS with the following exception: participants could contact study investigators should they wish to receive the results of their DBS. The consent form included local study investigators’ contact information.

The standard operating procedures used for the Dried Blood Spot (DBS) collection was adapted from WHO Manual for HIV Drug Resistance Testing Using Dried Blood Spot Specimens, March 2010.^3^

Below, we outline the DBS sample collection standard operating protocol in detail.

**Collecting DBS at the Interview Site**

1. Collect a DBS specimen only if the respondent is unable to provide a venous blood sample at the interview site.
2. Affix two Respondent ID Number labels onto the filter paper card.
3. Following a fingerprick, allow a large drop of free flowing blood to collect at the puncture site.
4. Working quickly, hold the filter paper by the edges and touch the filter paper gently against the large drop of blood and in one step allow a sufficient quantity of blood to soak through and completely fill or saturate a circle. A completely saturated spot will contain approximately 100 µL of blood.
5. Repeat until enough blood has been collected to fill at least 3 circles on the filter paper card.
6. Place DBS card in a suspended horizontal positive on a drying rack.
7. Dry DBS card horizontally for at least 4 hours, preferably overnight, depending on the ambient temperature and humidity.
8. To prepare for transport to the laboratory, gently place the DBS card into a paper folder, then into an envelope with other individually wrapped DBS cards. Make sure DBS cards do not come into direct contact with one another inside the envelope.
9. Upon arrival in the laboratory, unwrap DBS card and transfer into the Dessivac chamber, along with 3 large dessicant packets and a humidity indicator cards. Pump out air.
10. Once dry, the spots will appear a uniform dark brown with no areas of red coloration.

| Tips for collecting DBS |
| --- |
| - Do not press the filter paper against the puncture site. - Apply blood to only one side of the filter paper. - If the participant is a poor bleeder, apply blood onto different spots within the same collection circle to fill the circle but be sure not to overlay successive drops of blood on top of each other. - Do not “milk” the finger as excessive milking or squeezing the puncture site might cause hemolysis of the specimen or result in collection of tissue fluids with the specimen, which might adversely affect the test results. - Only one respondent’s blood may be collected on one card. |

**Preparing DBS from Venous Blood Specimen in the Laboratory**

1. EDTA anti-coagulated venous blood spotted onto filer paper as soon as possible after collection, and preferably within 24 hours of collection. Between the times of collection and spotting, blood should be stored at room temperature.
2. Affix two Respondent ID Number labels onto the filter paper card. Each filter paper card should only be spotted with the blood of a single respondent.
3. Invert the BD Vacutainer EDTA tube 2-3 times to mix the whole blood. Carefully open the blood collection tube.
4. Hold the filter paper by the edges. Using a micro-pipettor, aspirate 100 µL of whole blood. Without touching the pipette tip to the filter paper, dispense blood to the centre of one circle to fully saturate the circle.
5. Repeat until each circle on the filter paper card is filled.
6. Place DBS card in a suspended horizontal positive on a drying rack.
7. Dry DBS card horizontally for at least 4 hours, preferably overnight, depending on the ambient temperature and humidity.
8. After 4 hours if DBS card is not yet dry, transfer DBS card to the rack inside the Dessivac chamber, along with 3 large dessicant packets and a humidity indicator cards. Pump out air.
9. Once dry, the spots will appear a uniform dark brown with no areas of red coloration.

| Tips for drying DBS |
| --- |
| - Avoid touching or smearing the blood spots. - Keep away from direct sunlight. Care should be taken to avoid exposing DBS to environmental conditions that may compromise the integrity of the specimen. DBS should not be dried near an open window as sunlight, dust and in some cases flying insects may come in contact with the DBS during the drying procedure. - Do not use an external heat source to dry DBS. - Do not stack or allow DBS to touch other surfaces during the drying process. - Humidity indicator cards and dessicant packets have a colour indicator which changes from blue to pink as humidity increases. Dessicant packets can become moist after use with DBS, and also after storage in a humid environment. Store dessicant packets with humidity indicator cards to evaluate whether their moisture level has become too high. - Humidity indicator cards and dessicant packets can be recharged by heating at 50°C-60°C for 3-4 hours in a drying oven (*NB:* a longer drying may be needed if dessicant packets are moist). Cool for 10 minutes before returning both to storage in sealable Bitran gas impermeable plastic bag. |

**Packaging DBS**

1. Ensure DBS are completely dry before packing.
2. Affix a Respondent ID Number label onto a coin envelope that matches the ID Number on the DBS card.
3. Check the humidity indicator card inside the Dessivac chamber to ensure it is blue.
4. Open the Dessivac chamber and check that the DBS have turned a uniform dark brown.
5. Holding the DBS card by the edges, gently slide it into the coin envelope with the matching Respondent ID Number.
6. Place 5 DBS envelopes inside one Bitran gas-impermeable plastic bag along with 3 small dessicant packets and a humidity indicator card. Make sure the humidity indicator card is visible and can be easily read from outside the Bitran bag.
7. Gently press the partially sealed Bitran bag to expel air before sealing it completely.

**Handling and Storing DBS**

1. Bitran bags containing DBS envelopes should be kept in a -80°C freezer for storage.
2. DBS should only be taken out of cold storage when they are being tested or when dessicant packets and humidity indicator cards are being replaced.
3. When changing dessicant packets and humidity indicator cards in specimen bags that have been stored in a freezer, it is important to equilibrate the specimen bag to room temperature before opening the bag. Opening DBS bags immediately upon transfer from cold storage will result in condensation on the DBS specimens and Bitran bags.

**Supplementary Digital Content 3: Covariate Definitions**

To identify determinants of recent HIV testing among adolescent girls and young women who socialize at sex work hotspots, we focused on socio-demographic, health-system engagement, sexual behaviour, and risk perception.

For socio-demographic characteristics, we included the type of hotspot from where the participant was recruited (physical establishments [bars, night clubs, hotels, guest houses, lodges, restaurants, local brew dens, sex dens and brothels]; public spaces [streets and other public places such as beach, park etc.]; age at time of interview (14-18; 19-24 years); educational attainment (did not complete primary school; completed primary school; completed secondary school or higher) and currently receiving formal education. For health-system engagement, we included history of pregnancy; treatment for a bacterial sexually transmitted infection in the previous year; and engagement with sex worker programmes (awareness of an HIV programme, ever contacted by peers/staffs from a non-governmental organization/community-based organization, registration with HIV-prevention programme) as these questions were asked of all study participants. Within sexual behavior and risk perception, we included duration of sexual activity (< 2 years; >= 2 years); duration of sex work (< 2 years; >= 2 years) among women who sell sex (YSW) only; self-assessed risk of HIV acquisition for those who were not diagnosed with HIV (no risk at all/small/unsure; moderate/great).

**Supplementary Digital Content 4: Details of Ethical Review and Referral^4^**

Ethical approval was obtained from the Human Research Ethics Board at the University of Manitoba in Canada (HS16557 [H2013:295]), the Kenyatta National Hospital/University of Nairobi Ethics and Research Committee (P497/10/2013), and the Research Permit Committee of the National Commission for Science, Technology and Innovation, in Kenya. Participants provided written, informed consent prior to participation in study. The rationale for including participants from age 14 years onwards was the early age at which female sex workers reported selling sex for the first time^5^ and thus the importance of measuring vulnerabilities and risks experienced during the early period of sex work. The Transitions study was approved by the above ethics review committees to obtain written, informed consent for participation directly from individuals age 14 years and above without the consent of participant’s guardians because participants were considered mature minors. The rationale for defining the study population as mature minors included the following: to ensure confidentiality and access to HIV testing and counselling services, the consent procedure for Transitions was in accordance with the Kenya National HIV testing policies which supports HIV testing and counselling to “all or most adolescents under several conditions including showing maturity, of reproductive age, married, pregnant, or engaged in high-risk behavior.”^6^ Furthermore, the Transitions study uniquely sampled sexually active individuals who frequented sex work venues (hotspots) and did not sample households; eligible participants may live independently or on the streets and often do not have a legal guardian. Where a legal guardian is present, the guardians may be unaware of participants sexual behaviour and requiring guardian consent may potentially place participants at greater risk of further isolation or harm.  As per the Transitions study protocol, participants (and those who declined to participate) were referred to local programmes and clinics, including programmes that tailored services for adolescent girls and young women and those that provided services for female sex workers.

Participants who reported experience of any violence were referred to the Gender Based and Violence Recovery Centre at the Coast Provincial (referral) Hospital which is a joint public-private partnership between the Ministry of Health and International Centre for Reproductive Health Kenya.”

1. Cheuk E, Isac S, Musyoki H, et al. Informing HIV Prevention Programs for Adolescent Girls and Young Women: A Modified Approach to Programmatic Mapping and Key Population Size Estimation. *JMIR Public Health Surveill.* 2019;5(2):e11196.

2. National AIDS & STI Control Programme. *The Kenya HIV Testing Services Guidelines.* Nairobi, Kenya2015.

3. WHO. *WHO Manual for HIV Drug Resistance Testing Using Dried Blood Spot Specimens.* 2010.

4. Roberts E, Ma H, Bhattacharjee P, et al. Low program access despite high burden of sexual, structural, and reproductive health vulnerabilities among young women who sell sex in Kenya. *BMC Public Health (preprint).* 2020.

5. Musyoki H, Kellogg TA, Geibel S, et al. Prevalence of HIV, sexually transmitted infections, and risk behaviours among female sex workers in Nairobi, Kenya: results of a respondent driven sampling study. *AIDS Behav.* 2015;19 Suppl 1:S46-58.

6. National AIDS and STI Control Programme. *The Kenya HIV Testing Services Guideline.* National AIDS and STI Control Programme;2015.

**Supplementary Digital Content 5:**

| **Table 1A. Characteristics of the study participants aged 14-24 years by engagement in sex work in Mombasa, Kenya (N = 1299).** | | | | |
| --- | --- | --- | --- | --- |
| **Characteristics**  **(N (%))** | **Overall**  **(N = 1299)** | **YFSS**  **(N = 408)** | **YFNS**  **(N = 891)** | **p-value** |
| **Socio-demographic characteristics** |  |  |  |  |
| **Type of recruitment hotspot** |  |  |  |  |
| Physical establishments^a^ | 1069 (82.0%) | 348 (85.3%) | 721 (80.9%) | 0.06 |
| Public spaces^b^ | 230 (18.0%) | 60 (14.7%) | 170 (19.1%) |  |
| **Age in years** |  |  |  |  |
| 14-18 | 522 (40.0%) | 117 (28.7%) | 405 (45.5%) | < 0.001 |
| 19-24 | 777 (60.0%) | 291 (71.3%) | 486 (54.5%) |  |
| **The highest education level** |  |  |  |  |
| Did not complete primary school | 305 (23.5%) | 124 (30.4%) | 181 (20.3%) | < 0.001 |
| Completed primary school | 671 (51.7%) | 210 (51.5%) | 461 (51.7%) |  |
| Completed secondary school or higher | 323 (24.9%) | 74 (18.1%) | 249 (27.9%) |  |
| **Currently receiving formal education** | 266 (20.5%) | 33 (8.1%) | 233 (26.2%) | < 0.001 |
| **Health-system engagement** |  |  |  |  |
| **Ever pregnant** | 493 (38.0%) | 234 (57.4%) | 259 (29.1%) | < 0.001 |
| **Treated STI last 1 year** | 223 (17.0%) | 91 (22.3%) | 132 (14.8%) | 0.001 |
| **Programme engagement** |  |  |  |  |
| Not aware of HIV services | 1052 (81.0%) | 303 (74.3%) | 749 (84.1%) | < 0.001 |
| Awareness of HIV services | 127 (9.8%) | 47 (11.5%) | 80 (9%) |  |
| Ever contacted by peers/staff from an NGO/CBO | 56 (4.3%) | 21 (5.1%) | 35 (3.9%) |  |
| Registered with NGO/CBO | 64 (4.9%) | 37 (9.1%) | 27 (3%) |  |
| **Ever received an HIV test** | 1121 (86.0%) | 383 (93.9%) | 738 (82.8%) | < 0.001 |
| **Tested for HIV in the last 1 year^c^** | 924 (72.0%) | 345 (85.4%) | 579 (65.4%) | < 0.001 |
| **Last HIV testing location** |  |  |  |  |
| Public/government facility | 989 (88.0%) | 355 (92.7%) | 634 (85.9%) | 0.008 |
| NGO/CBO through outreach | 41 (4.0%) | 10 (2.6%) | 31 (4.2%) |  |
| Private facility | 22 (2.0%) | 4 (1.0%) | 18 (2.4%) |  |
| Other/Do not recall | 69 (6.0%) | 14 (3.7%) | 55 (7.5%) |  |
| **Sexual behavior and risk perception** |  |  |  |  |
| **Duration of sexual activity^d^** |  |  |  |  |
| <2 years | 434 (33.4%) | 63 (15.4%) | 371 (41.6%) | < 0.001 |
| >=2 years | 865 (66.6%) | 345 (84.6%) | 520 (58.4%) |  |
| **Duration in sex work** |  |  |  |  |
| <2 years | 200 (49.0%) | 200 (49.0%) | -- |  |
| >=2 years | 208 (51.0%) | 208 (51.0%) | -- |  |
| **Self-assessed risk of HIV acquisition^e^ (N=1283)** |  |  |  |  |
| No risk at all/small/unsure | 745 (58.0%) | 222 (55.1%) | 316 (35.9%) | < 0.001 |
| Moderate/Great | 538 (42.0%) | 181 (44.9%) | 564 (64.1%) |  |

Abbreviations: CBO (community-based organization); NGO (non-governmental organization); YFNS (young females who do not sell sex); STI (sexually transmitted infection); YFSS (young females who sell sex)

^a^Physical establishments hotspots include bars, night clubs, hotels, guest houses, lodges, restaurants, local brew dens, sex dens and brothels

^b^Public spaces hotspots include streets and other public places

^c^Excluding individuals who were diagnosed with HIV >1 year ago

^d^N=55/1299 missing was imputed by adjusting for age at the interview

^e^Excluding individuals who disclosed they are living with HIV

**Number of newly diagnosed HIV among YFSS at hotspots**

**Rapid test acceptance**

**&**

**sensitivity**

A. Number of YFSS who frequent hotspots^1^:

6,127 (range 4,793-7,462)

**Potential**

**Feasible**

**452 (range: 193-881)**

**313 (range: 134-610)**

**100%**

**&**

**100%**

**86.1%**

**&**

**80.4%**

B. HIV prevalence among YFSS who frequent hotspots:

10.1% (95% CI: 7.2-13.7)

C. Undiagnosed HIV among YFSS who frequent hotspots:

73.0% (95% CI: 55.9-86.2)

A * B * C

D. Number of YFSS who frequent hotspots and are living with HIV and unaware of HIV status:

452 (range: 193-881)

**Figure 2A. Triangulating the number of young women who sell sex living with HIV who could be diagnosed via hotspot-based HIV testing strategy in Mombasa, Kenya.**

Abbreviation: YFSS (young females who sell sex).

^1^Cheuk E, Isac S, Musyoki H, et al. Informing HIV Prevention Programs for Adolescent Girls and Young Women: A Modified Approach to Programmatic Mapping and Key Population Size Estimation. JMIR Public Health Surveill. 2019;5(2):e11196.

**Number of newly diagnosed HIV among YFNS at hotspots**

**Rapid test acceptance**

**&**

**sensitivity**

A. Number of YFNS who frequent hotspots^1^

9,508 (range 7,379-11,635)

**Potential**

**Feasible**

**240 (range: 93-506)**

**175 (range: 68-369)**

**100%**

**&**

**100%**

**90.7%**

**&**

**80.4%**

B. HIV prevalence among YFNS who frequent hotspots:

3.6% (95% CI: 2.5-5.1)

C. Undiagnosed HIV among YFNS who frequent hotspots:

70.0% (95% CI: 50.6-85.3)

A * B * C

D. Number of YFNS who frequent hotspots and are living with HIV and unaware of HIV status:

240 (range: 93-506)

**Figure 3A. Triangulating the number of young women not engaged in sex work living with HIV who could be diagnosed via hotspot-based HIV testing strategy in Mombasa, Kenya.**

Abbreviation: YFNS (young females who do not sell sex).

^1^Cheuk E, Isac S, Musyoki H, et al. Informing HIV Prevention Programs for Adolescent Girls and Young Women: A Modified Approach to Programmatic Mapping and Key Population Size Estimation. JMIR Public Health Surveill. 2019;5(2):e11196.

**Rapid test acceptance**

**&**

**sensitivity**

**Number of newly diagnosed HIV among AGYW at hotspots**

A. Number of AGYW who frequent hotspots^1^

15,635 (range 12,172 – 19,097)

**Potential**

**Feasible**

**510 (range: 238-925)**

**366 (range: 171-664)**

**100%**

**&**

**100%**

**89.3%**

**&**

**80.4%**

B. HIV prevalence among AGYW who frequent hotspots:

5.6% (95% CI: 4.3-6.9)

C. Undiagnosed HIV among AGYW who frequent hotspots*:

58.2% (95% CI: 45.5-70.2)

A * B * C

D. Number of AGYW who frequent hotspots and are living with HIV and unaware of HIV status:

510 (range: 238-925)

**Figure 4A. Triangulating the number of adolescent girls and young women living with HIV who could be diagnosed via hotspot-based HIV testing strategy in Mombasa, Kenya.**

Abbreviation: AGYW (adolescent girls and young women).

*Sensitivity analysis: based on the assumption that participants who were not willing to disclose their HIV-status were living with HIV and were aware of their status

^1^Cheuk E, Isac S, Musyoki H, et al. Informing HIV Prevention Programs for Adolescent Girls and Young Women: A Modified Approach to Programmatic Mapping and Key Population Size Estimation. *JMIR Public Health Surveill.* 2019;5(2):e11196.
